# Factors influencing second language learning based on the research of Lightbown and Spada

**DOI:** 10.3389/fpsyg.2024.1347691

**Published:** 2024-02-06

**Authors:** Chuandai Qiao

**Affiliations:** School of Foreign Languages, Hubei University of Automotive Technology, Shiyan, China

**Keywords:** Lightbown and Spada, motivation, aptitude, personality, intelligence, learner preferences

## Abstract

Deep insights into the factors influencing second language learners can positively impact learners’ self-intervention and guide language teachers in selecting appropriate teaching materials and strategies. Drawing from Lightbown and Spada’s framework encompassing motivation, aptitude, personality, intelligence, and learner preferences, this paper examines the relationship between individual learners’ characteristics and second language learning effects across five aspects by dialectically considering the research methods suitable for different learner variables. By incorporating recent research and offering practical implications, this paper has the potential to contribute valuable insights to both researchers and practitioners in the field of language education.

## 1 Introduction

The dynamic and intricate nature of second language acquisition involves a complex interrelation between individual physiological and psychological traits and the resulting outcomes of language acquisition (Alanen, 2003; Dörnyei, 2013; Dörnyei, 2014; Banaruee et al., 2023a,b). Individuals’ anticipation of success in second language acquisition can be gauged to some extent by considering information on their personalities, specific intellectual abilities, motivation, or age (Candlin and Mercer, 2001). Lightbown and Spada assert that these beliefs typically stem from anecdotal evidence, often derived from personal experiences. Therefore, the primary objective of their study is to examine the validation of anecdotal evidence through research findings. They listed 12 typical characteristics contributing to language learning and organized them into five categories: intelligence, aptitude, motivation, personality, and learner preferences (Lightbown and Spada, 2000).

Lightbown and Spada acknowledge the intricate interplay of various factors influencing frustration in second language learners. Consequently, by amalgamating diverse scholars’ perspectives and research methodologies, they offer critical insights into the limitations and challenges inherent in studying the factors influencing second language acquisition. Based on their study, this review article amalgamates recent research advancements and provides recommendations to alleviate the concerns they highlighted to contribute valuable insights to both researchers and practitioners within the domain of language education.

## 2 Factors influencing second language learning

### 2.1 Intelligence

Relying on Genesee’s empirical data from 1976 regarding the connection between intelligence and language sub-skills, Lightbown and Spada suggested that intelligence, as assessed through verbal IQ tests, plays a significant role in learning tasks related to language analysis and rules, such as reading and grammar. However, when it comes to spoken language emphasizing communication and interaction, the impact of intelligence is less apparent (Lightbown and Spada, 2000).

While the notion that intelligence factors significantly influence logical and rule-like language skills, such as reading and grammar is widely embraced within the academic community (Jimenez et al., 2003; Maftoon and Sarem, 2012; Salehi and Sadighi, 2012), some scholars assert that the verbal IQ test employed by Lightbown and Spada possesses certain limitations (Chowdhury, 2010; Kakhramonov, 2020). Consequently, these researchers utilize non-verbal intelligence tests to gauge participants’ IQ, arriving at conclusions akin to those by Lightbown and Spada. Furthermore, their investigations into how IQ factors impact language skills, such as reading and grammar, have substantially broadened the depth of the field. Nonetheless, the diverse range of intelligence criteria challenges traditional intelligence (IQ) tests in capturing the full complexity of intellect (Ellis, 1994). Consequently, the research on the impact of intelligence on second language acquisition remains controversial. Some studies have also indicated that students without high IQ levels have succeeded in second-language learning (Brown, 2014).

Given the intricate nature of intelligence and the potential drawbacks associated with traditional measures, Gardner’s “Multiple Intelligence Model” has garnered increasing attention from researchers. Departing from the conventional perspective that confines intelligence to cognitive abilities linked to verbal and numerical skills, Gardner expanded the concept to encompass linguistic, logical-mathematical, spatial, musical, bodily-kinesthetic, intrapersonal, interpersonal, and naturalistic intelligence (Gardner, 2008). This multifaceted approach empowers students by recognizing diverse forms of intelligence to foster their confidence. As intelligence manifests in various ways, each individual possesses a unique combination of these intelligences, offering valuable insights into studying learner preferences.

### 2.2 Aptitude

The aptitude for second language (L2) learning is characterized by the strengths exhibited by individual learners in cognitive abilities pertinent to information processing during L2 acquisition. This encompasses their performance in diverse contexts and at different stages of the learning process, in comparison to the broader population (Harley and Hart, 1997; Robinson, 2005). Drawing on the language learning experience of CJ, a boy with exceptional language talents in Au et al., (1989) case study, Lightbown and Spada suggested that learning quickly stands out as a distinctive characteristic of aptitude.

Within the framework of the Modern Language Ability Test (MLAT) and Pimel Language Ability Test (PLAR), the assessment criteria for language ability predominantly hinge on vocabulary retention and comprehension of grammatical rules. While this proficiency was validly assessed in the early stages of grammar translation or audiolingual methods, the shift toward communicative teaching methods has led educators and researchers to observe that the capacity to recognize and memorize new sounds may transition from an advantage to a limitation in meaning-oriented instruction (Lightbown and Spada, 2000). In recent years, a growing number of scholars have undertaken a thorough examination of the relationship between aptitude and second language acquisition (Skehan, 2012). Their discussions span various perspectives, investigating the content and characteristics of aptitude, along with its associations with factors like age, instructional methods, and other pivotal aspects of language learning, including the distinction between explicit and implicit knowledge (Robinson, 2005; Doughty and Mackey, 2021; Li and Zhao, 2021). Among these factors, investigating age and learning ability has consistently been a focal point of empirical research in this field. Researchers such as Birgit Harley and Doug Hart observed a positive correlation between second language outcomes and memory in early immersion second language acquisition, particularly in first-grade students. For late (adolescent) second language outcomes, they noted a positive correlation with the analytical dimension of language ability (Harley and Hart, 1997). ShaoFeng Li’s research revealed that high school students were more prone to draw on aptitude than university students. Additionally, aptitude correlated more strongly with explicit treatments than implicit treatments in the language learning context (Li and Zhao, 2021). These empirical studies build upon Lightbown and Spada’s earlier hypothesis, suggesting that language ability, as assessed by traditional aptitude tests, comprises a set of cognitive abilities that play a more prominent role in the initial stages of second language development and conscious learning conditions (Candlin and Mercer, 2001; Li and Zhao, 2021).

Nevertheless, ongoing debates persist in the realm of competence and second language acquisition. Key points of contention include whether aptitude represents the “upper limit” of language learning and whether the components of aptitude play distinct roles in various learning stages of second language learners, such as the beginning and proficiency stage. These unresolved issues warrant further research and exploration (Kakhramonov, 2020; Doughty and Mackey, 2021).

### 2.3 Motivation

Motivation for second language learning is a multifaceted phenomenon that defined by two key factors: learners’ communicative needs and attitudes toward the second language community. Lightbown and Spada argued that an individual’s identity and social dynamics, including power relationships, significantly influence language motivation. Both children and adults are sensitive to these social dynamics and power relationships, which can impact their motivation in the language-learning process (Lightbown and Spada, 2000). Lightbown and Spada’s perspectives are influenced by social cognitive theory, where motivation has consistently played a prominent role from early modeling studies to its contemporary conceptualization involving agencies. Within the profound integration of second language acquisition and social cognitive theory, various theoretical frameworks for second language learner motivation have emerged, including attribution theory (Weiner, 1972), self-efficacy theory (Bandura, 1977), the L2 motivation self-system (Dörnyei and Taguchi, 2009), and self-determination theory (McEown and Oga-Baldwin, 2019).

Lightbown and Spada made another significant contribution by integrating theories from educational psychology into the realm of second language acquisition. They examined the interplay between teachers’ classroom instruction and motivation in the second language classroom, proposing that teachers should diversify activities, tasks, and materials. This approach involves the adoption of teaching strategies like cooperative and non-competitive goals to enhance students’ motivation for learning (Lightbown and Spada, 2000). However, Lightbown and Spada’s research primarily concentrated on the impact of external factors, such as the social environment and teachers. The self-model of second language acquisition still needs to be explored in their work. Dörnyei’s latest study specifically addresses this question, delving into the self-model and its role in second language acquisition motivation (Dörnyei, 2013). By refining and expanding the psychological theory of motivation, he introduced a groundbreaking conceptual framework called the bilingual motivational self-system. This model utilizes a process-oriented approach to scrutinize the various stages of motivation. Notably, the new motivational model displays an apparent inclination toward personality psychology, representing a shift in perspective on the nature of motivation from external behaviors to internal cores (Dörnyei and Ushioda, 2009; Dörnyei, 2014). Furthermore, the model successfully addresses the challenge posed by the absence of a language community in society and offers a fresh perspective on studying motivation. It serves as a framework that can delineate both the starting and ending points of motivational behavior by referencing both authentic and possible selves (Dörnyei, 2020). Concerning motivation assessment, Gardner and Lambert introduced the synthesis dimension and tool dimension, developing a motivation assessment tool named the Attitude/Motivation Test Battery (AMTB). This tool has profoundly influenced on subsequent research in the field (Gardner, 2008).

### 2.4 Personality

While some linguists and psychologists argue that personality plays a significant role in the success of second language acquisition, identifying and measuring personality remains challenging (Lalonde and Gardner, 1984; Robinson et al., 1994; Novikova et al., 2020). Personality is often intertwined with other factors influencing second language acquisition, complicating the determination of its specific impact. Lightbown and Spada highlighted two primary challenges in personality research affecting progress. Firstly, there needs to be more consensus on the relationship between personality and language success. Some studies suggest that individuals with an extroverted personality may be more likely to succeed due to the self-confidence and adventurous spirit required. At the same time, other research indicates that many successful language learners do not necessarily exhibit high levels of extroversion. Secondly, the measurement and evaluation criteria for language learning success need to be more consistent. In the realm of language acquisition, researchers delving into communicative competence may wield criteria that differ from those who focus on grammatical accuracy or metalinguistic knowledge, and this variance in measurement standards can potentially engender confusion when assessing success in language acquisition (Lightbown and Spada, 2000).

Advancements in modern medicine and neurolinguistics have begun to shed light on the challenges previously encountered in understanding the role of personality in second language acquisition. Researchers such as Grzegorz Dogil and Susanne Maria Reiterer employed functional magnetic resonance imaging to observe differences in brain activity among individuals with varying linguistic abilities during phonological tasks, including phonological differential perception, imitation, and reading (Dogil and Reiterer, 2009). David Robinson and Norman Gabriel utilized the Eysenck Personality Questionnaire (EPQ) to investigate the connection between personality traits and language learning abilities. Their findings indicated that individuals with high neuroticism and high extraversion scores tended to perform better on oral tests than on written tests. In contrast, those with high neuroticism and low extraversion scores showed better performance on written tests than on verbal tests (Robinson, et al., 1994). While results from these studies may still vary, the interdisciplinary research perspectives undeniably offer more possibilities for addressing the complexities of these issues.

### 2.5 Learner preferences and styles

Individuals demonstrate a range of learning preferences and styles, advancing at different rates owing to inherent biological and psychological distinctions (Reiff, 1992). Based on learners’ learning characteristics in a specific domain, Lightbown and Spada categorized them as “visual,” “aural,” “and “kinaesthetic” (Lightbown and Spada, 2000). Because learning styles are multidimensional, researchers have devised various instruments to assess and measure these diverse learner preferences (Dunn and Dunn, 1972; Schmeck et al., 1977). Among these instruments, field independence (FI)/field dependence (FD), which relates to how individuals perceive and memorize information (Kheirzadeh and Kassaian, 2011), has been the subject of extensive investigation. Furthermore, an increasing number of researchers have focused on studying the alignment of learner styles with teaching styles and learning strategies (Ehrman and Oxford, 1990). Aligning students’ learning styles with appropriate teaching approaches can significantly enhance their motivation, performance, and achievements (Brown, 1973). Another noteworthy observation is that adult second language learners frequently articulate their learning beliefs more explicitly than their learning styles (Gregorc, 1979). The acknowledged mediating role of learner beliefs in the classroom further underscores their significance. Additionally, when these beliefs (or metacognitive knowledge) act as mediators, they possess the capacity to influence both learners and teachers, shaping their behavior (Alanen, 2003; Banaruee et al., 2022).

As observed by Lightbown and Spada, investigations into learner beliefs have traditionally leaned heavily on quantitative and descriptive research methods. Nevertheless, a discernible shift emerged in the 2000s, witnessing a pronounced inclination towards a qualitative approach. Researchers during this period exhibited a proclivity for embracing a contextual perspective in their exploration of learner beliefs (Barcelos and Kalaja, 2011). Situated within the socio-cultural framework, some scholars meticulously investigate the intricate mechanisms and trajectories that characterize the transformation between learners and their educational milieu (Negueruela-Azarola, 2011). Findings underscore that educators wield substantial influence in enhancing students’ adaptability to second language acquisition through the judicious deployment of varied pedagogical strategies and the establishment of an optimal learning environment (Reiff, 1992; Sims and Sims, 1995; Peng, 2011).

### 2.6 Other factors

In contrast to the intricate task of defining and measuring various factors that impact second language acquisition, the role of age as an explanatory factor for differences in second language acquisition benefits from more accessible definitions and measurement methods. Since the introduction of Lenneberg’s Critical Period Hypothesis (CPH) for first language (L1) acquisition in 1967, numerous researchers have delved into investigating the impact of age factors on various language groups and language skills (Patkowski 2013; Muñoz, 2014). There has been a prevalent assumption that young children predominantly depend on memory-based processes, while adults are more notably characterized by rule-based learning (Nikolov and Djigunović, 2006). The reduction in procedural memory for language compels adults learning a second language to depend on explicit learning, leading to the engagement of a cognitive system distinct from the one supporting their native language (Paradis, 2004). Nevertheless, the adverse impacts of phonological difficulties stemming from missed critical periods and diminished memory in adult learners may not be decisive factors for success in second language acquisition. Learners’ motivation to acquire a second foreign language and their aspiration to integrate into the social life of the target language can mitigate the negative effects associated with age (Widyaningsih et al., 2022). Some recent studies are introducing innovative approaches to interindividual variation from a neurocognitive perspective. Their research delves into the intricate relationship between the cognitive levels of second language learners and their age as well as second language proficiency (Faretta-Stutenberg, 2023; Fromont, 2023).

Lightbown and Spada’s study neglected to account for the impact of culture on second language acquisition. Language is not merely a product of culture, it also serves as a symbol of culture, which establishes an intrinsic connection between language and literature (Gleason, 1955). In the design of language courses, it is crucial for teachers to consider cultural diversity, employ suitable teaching strategies, and harness the enriching tension arising from cultural differences to enhance language learning (Tseng, 2002; Kuo and Lai, 2006). Currently, there is a growing emphasis on investigating the impact of the socio-cultural background of both teachers and learners on the language learning process. Enhancing the cultural awareness of language learners and educators has emerged as a crucial focal point in contemporary research (Banaruee et al., 2023a,b).

The findings indicate that when learners actively engage with the culture associated with the language they are acquiring, it significantly enhances their academic performance and fosters a more profound understanding of the language (Arabski and Wojtaszek, 2011; Pourkalhor and Esfandiari, 2017).

## 3 Implications and suggestions for further research

Despite certain limitations inherent in Lightbown and Spada’s study on the factors influencing second language acquisition, I contend that it retains its illuminating value for contemporary research, particularly in the following three facets. Initially, Lightbown and Spada astutely observed that certain researchers were oblivious to distinctions in various behavioral characteristics. They employed identical labels to depict dissimilar behavioral traits or haphazardly scrutinized factors that could not be directly observed and measured through questionnaires (Lightbown and Spada, 2000). They exemplified this issue with motivational studies with the aim of cautioning researchers about potential pitfalls in subsequent investigations. According to them, deducing the relationship between individual characteristics and language learning from a questionnaire or a single variable is challenging. Firstly, learner variables interact in intricate ways, and certain traits, like motivation and extraversion, are interdependent and intricate. This makes direct observation and measurement challenging. Secondly, individual elements exert distinct effects and responses to specific aspects of language skills. For instance, highly motivated learners excel in informal situations but may lag behind in meta-linguistic knowledge. Furthermore, the relationship between learners’ characteristics and language learning outcomes does not always imply a causal connection. Considering high motivation levels contributing to a successful language learner, a concept supported by Gardner and Lambert who argued that elevated motivation in a formal learning environment could predict a learner’s effectiveness (Gardner et al., 1989). Nonetheless, ascribing an individual’s success in language acquisition exclusively to motivation may be precipitous, given the potential oversight of other contributory factors (Li and Wang, 2018). Lightbown and Spada conduct a thoughtful and dialectical analysis of individual learner characteristics and their influence on the effectiveness of second language learning. They emphasize the nuanced understanding that learner variables interact in complex ways, presenting a comparative analysis of issues in existing research. This provides valuable insights for future investigations.

Secondly, Lightbown and Spada highlight distinctions between factors influencing the effectiveness of second language acquisition, such as intelligence and motivation, and various language skills, such as reading ability and oral communication proficiency. They challenge the unidimensional claim that success in one language skill defines overall success in second language acquisition. This stance, they argue, can yield perplexing and even contradictory research results. For instance, in an informal language learning setting, motivated learners might excel if aptitude tests focus on measuring oral communication skills. However, in other studies, highly motivated learners may not exhibit greater success if the test primarily assesses language knowledge. Lightbown and Spada’s research have paved the way for empirical studies investigating the correlation between diverse influencing factors and various language skills.

Finally, Lightbown and Spada adopt an interdisciplinary perspective, providing a comprehensive review and critical reflection on various factors and research methods influencing second language acquisition. Their research not only offers valuable insights to expand the study of factors influencing second language acquisition but also highlights certain bottlenecks and challenges within this field. Nevertheless, the challenges raised by Lightbown and Spada have found innovative solutions through the integration of current developments in other disciplines. For example, in the realm of motivation, Dörnyei draws on concepts from personality psychology and social psychology to reexamine integrative motivation, introducing a new conceptual framework—the bilingual motivational self-system (Dörnyei, 2013, 2014). By amalgamating Lightbown and Spada’s research with advancements by other scholars on pertinent issues, this study not only delves into the highlights and obstacles in their work but also provides insights for interpreting these challenges and bottlenecks from an interdisciplinary perspective.

## Author contributions

CQ: Writing – original draft.
